# Use of Scores in Risk Stratification of Febrile Neutropenia—A Scoping Review

**DOI:** 10.3390/cancers18060987

**Published:** 2026-03-18

**Authors:** Alexander Djupnes Fuglkjær, Frederik Christensen, Deniz Kenan Kılıç, Laurids Østergaard Poulsen, Paw Jensen, Carsten Utoft Niemann, Tarec Christoffer El-Galaly, Izabela Ewa Nielsen

**Affiliations:** 1 Department of Materials and Production, Aalborg University, 9220 Aalborg, Denmark; 2 Department of Oncology, Clinical Cancer Research Center, Aalborg University Hospital, 9000 Aalborg, Denmark; 3 Department of Clinical Medicine, Aalborg University, 2450 Aalborg, Denmark; 4 Department of Hematology, Clinical Cancer Research Center, Aalborg University Hospital, 9000 Aalborg, Denmark; 5 Department of Hematology, Copenhagen University Hospital, Rigshospitalet, 2100 Copenhagen, Denmark; 6 Department of Clinical Medicine, University of Copenhagen, 2200 Copenhagen, Denmark; 7 Department of Clinical Medicine, Aarhus University, 8200 Aarhus, Denmark; 8 Department of Molecular Medicine, Aarhus University Hospital, 8200 Aarhus, Denmark; 9 Department of Clinical Epidemiology, Aarhus University Hospital, 8210 Aarhus, Denmark; 10 Department of Hematology, Aarhus University Hospital, 8200 Aarhus, Denmark

**Keywords:** febrile neutropenia, risk stratification, scoping review, risk score, oncology, hematology

## Abstract

**Purpose:** A scoping review of the published risk stratification scores for febrile neutropenia (FN) was performed to provide a basis for further research and optimization of risk stratification models that can support evidence-based clinical decision-making with a combined individual patient and health resource perspective. **Methods:** The scoping review utilized the Preferred Reporting Items for Systematic Reviews and Meta-analysis extension for Scoping Reviews (PRISMA-ScR). Studies reporting risk stratification models for FN and published in the PubMed and/or Scopus databases between 2000 and 2024 were retrieved and reviewed. Study eligibility criteria were adult cancer patients and articles utilizing FN risk stratification methods. Two researchers reviewed all relevant studies separately to determine if they were eligible for inclusion and extracted the necessary data. **Results:** A total of 210 papers was screened by title and abstract. A further 158 were screened by retrieval and eligibility, and 14 studies were found eligible after reviewing full papers. Studies have different cohort sizes (min 31, max 4434), age and gender distributions, cancer types (1 hematological, 3 gynecological, 10 mixtures of hematological and solid cancers), definitions of FN and complication, study type (2 retrospective, 12 prospective). The resulting papers mostly focused on validating CISNE and MASCC scores. Additionally, they investigated possible improvements by evaluating revised versions of the MASCC score. **Conclusions:** The scoping review revealed inconsistencies in key definitions when using risk stratification scores. It is concluded that the field could benefit from more consensus in definitions and research approaches to secure the generalizability and utility of the research.

## Simple Summary

Febrile neutropenia is a frequent complication in cancer patients undergoing anticancer treatment. Decisions regarding outpatient management are often supported by risk stratification tools. This scoping review examines the utilization of these risk stratification tools. It analyzes their use in the current literature and discusses the pitfalls that validation studies should consider. It concludes that one of the most pertinent differences in the current literature lies in outcomes and inclusion/exclusion criteria, which are often modified to increase predictive performance. These methodological heterogeneities can make comparisons across validation studies particularly difficult. Therefore, a standardized approach to febrile neutropenia risk stratification should be considered to facilitate more reliable comparison and validation.

## 1. Introduction

Febrile neutropenia (FN) is a frequent complication in cancer patients undergoing antineoplastic treatment and is traditionally defined as fever in the presence of a reduced absolute neutrophil count (ANC) [1]. Neutrophils are critical to prevent bacterial and fungal infections, and a low neutrophil count increases the risk of serious infections [2,3]. A common dose-dependent side effect of chemotherapy is myelosuppression and consequently neutropenia with associated susceptibility to bacterial infections with potentially fatal outcomes. Among patients undergoing chemotherapy, FN is reported in 7.9–21.5% [4,5,6]. With an associated in-hospital mortality rate of approximately 10%, FN contributes substantially to mortality in this population of patients [6,7].

There is a substantial global shortage of healthcare resources, particularly healthcare professionals, which is further exacerbated by population aging and the resulting increase in cancer risk [8,9]. In addition, the improvement and availability of a growing number of cancer treatments have led to a growing population of cancer patients who are at risk of treatment-induced complications. Due to the unsustainable growth in resource demands, clinical practices need to adaptively assess the risk of patients, such that resources can be allocated to patients at the highest risk for complications. Since FN frequently leads to hospital admissions, it is also attributable to significant increasing costs and use of resources in cancer care. Besides this, patients with FN often require intensive monitoring and timely medical intervention, thereby occupying a large portion of beds in oncology and hematology wards [10,11,12]. All of which often results in a significant reduction in quality of life for the affected patient [13]. There exist methods of predicting FN in patients, such as the FENCE score introduced by Aagaard et al. [14]. However, it is not always clear which patients diagnosed with FN belong to the high-risk group and which belong to the low-risk group, and which belong to the low-risk group.

Therefore, accurate risk models that can identify high-risk FN patients that should be managed in-hospital versus low-risk FN patients that may appropriately be managed in an outpatient setting are highly relevant [15]. To identify patients with either high-risk or low-risk FN, several risk stratification scores have been developed. The current state-of-the-art is the Multinational Association for Supportive Care in Cancer (MASCC) [16] and the Clinical Index of Stable Febrile Neutropenia (CISNE) [17]. These scores are based on a series of clinicopathologic features of the patient; for example, the MASCC score utilizes features such as type of cancer, burden of illness, hypotension, and age, while the CISNE score includes factors like ECOG performance scores, history of cardiovascular disease, and presence of stress-induced hyperglycemia. These scores can help guide decisions on hospitalization, choice of antibiotics, and other aspects of management. The most commonly used models are MASCC and CISNE, but other models will also be included in this review to gain a deeper insight into the field.

As medical knowledge advances with possibilities to combine this with novel machine learning technologies due to the implementation of electronic health records, new decision support tools may be able to offer more accurate predictions based on a larger number of clinical variables. Machine learning has been introduced as a tool to risk stratify patients for specific medical events in other settings; for example, predicting the risk of in-hospital mortality of patients with heart failure in the ICU and risk for secondary cardiovascular disease prevention in multiethnic patients [18,19,20]. However, the methodologies described in these papers have not yet been widely applied to FN.

The purpose of this scoping review is to review the current literature to describe the use of risk stratification tools for FN, while also presenting potential areas lacking in the research. The review will serve as a basis for discussions of the gaps in the current literature and the possibilities for improvement in the field of risk stratification models for FN. By doing so, it seeks to grant a comprehensive overview of how scores for risk stratification of patients suffering from FN are utilized and how they can potentially be enhanced.

## 2. Methodology

This is a scoping review that aims to examine the scope, broadness, and nature of the literature, as well as identify potential knowledge gaps [21,22,23]. This review utilized the Joanna Briggs Institute (JBI) methodology for scoping reviews, which begins by specifying the aim and subsequently defines inclusion/exclusion criteria [21]. Identified studies were then analyzed and presented. The review was drafted and reported using the Preferred Reporting Items for Systematic Reviews and Meta-Analysis Extension for Scoping Reviews (PRISMA-ScR) checklist [24,25]. The protocol was not prospectively registered in PROSPERO or any other systematic review registry.

### 2.1. Search Strategy

The literature search was made using PubMed and Scopus and restricted to publications from 2000 to 2024. Database searches were conducted in advance to find all relevant evidence in the scope of FN and risk stratification. PubMed and Scopus queries are ‘((“febrile neutropenia”[Title/Abstract]) OR (“fn”[Title/Abstract])) AND “risk-stratification”[Title/Abstract]’ and ‘TITLE-ABS-KEY ((“febrile neutropenia” OR “FN”) AND “risk stratification”)’, respectively. The query was chosen to obtain a broad range of papers, while only focusing on papers using the term risk stratification.

### 2.2. Inclusion and Exclusion Criteria

The Population, Intervention, Comparison, Outcomes, and Study (PICOS) design framework was utilized for the eligibility criteria [26,27]. The inclusion criteria used in this review are given in Table 1.

The studies whose document type and language are not article and English respectively, were excluded. In addition, pediatric studies and unavailable full-text articles were left out. However, studies in which cohorts included patients below 18 years of age were included if the patients were treated within adult oncological treatment settings. In such cases, eligibility was determined based on treatment context rather than age.

### 2.3. Study Selection and Data Extraction

After removing duplicate records, titles, and abstracts of the identified papers, they were examined by two researchers (ADF and DKK), who then eliminated non-relevant papers based on the criteria presented in Table 1. Records that were retrieved for full-text screening were downloaded and stored. ADF and DKK removed the studies that did not meet the predetermined eligibility criteria. The chosen literature was cross-validated, and data extraction was executed by ADF and DKK. A third and fourth researchers, TCE and IEN, were consulted for decision-making in disagreement situations. Extracted data contains study details (author, year), cohort size, methods used for FN risk stratification, types of cancer, and definitions of complication and FN.

## 3. Results

**Figure 1:**
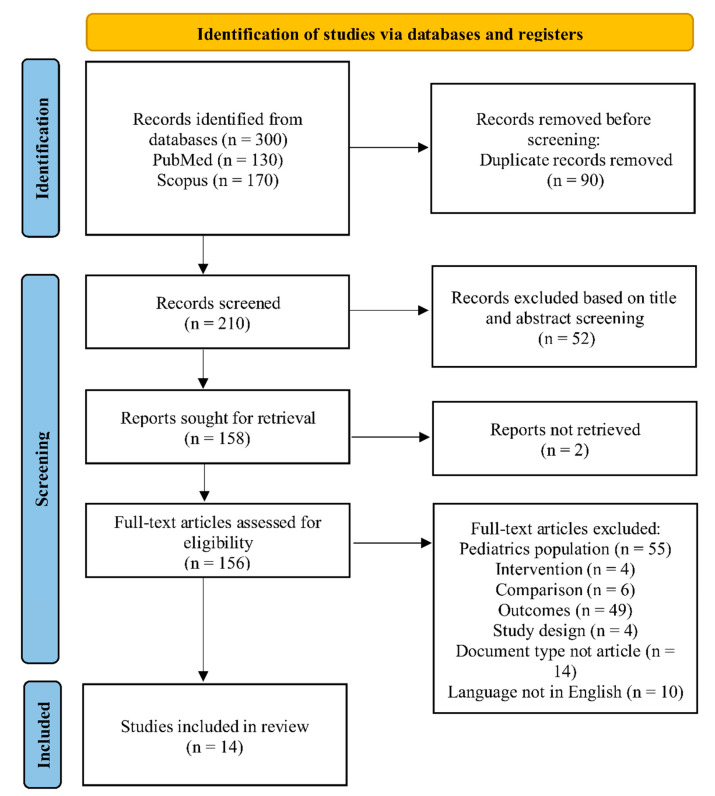
PRISMA statement flow diagram for literature search.

A PRISMA flow diagram in Figure 1 illustrates the study selection process. A total of 300 records were obtained from the PubMed and Scopus databases using the queries specified in the methodology section. Out of these, 90 duplicate records were eliminated, leaving 210 records for screening. After examining the titles and abstracts, 52 records were excluded based on the search strategy and predefined eligibility criteria. The full texts of two of the remaining records were not available. Finally, 142 studies were excluded during full-text article screening according to PICOS components, document type, and article language as given in Table 1. As a result, 14 articles were selected for inclusion in the study.

The results from examining a wide range of papers utilizing risk stratification of FN are shown in Table 2. The table describes the defining factors of the papers, such as cohort size, distribution of gender and cancer types, methods used, setting as well as definition of primary outcome and potential secondary outcome. These outcomes are to be understood as what the papers used to define a high-risk patient.

### 3.1. Size of Cohort and Cancer Type Distribution

The mean cohort size in this review was 486 patients with a standard deviation of 1104 patients. The very large standard deviation is caused by [29] being significantly larger than any other cohort size. This is further cemented by the fact that by removing [29], the mean and standard deviation were reduced to 182 and 149 patients, respectively.

Out of the 14 studies, one study focused on hematological patients [32], three studies solely focused on gynecological patients [28,37,38], and 10 studies concentrated on both hematological and solid cancers to varying ratios.

### 3.2. Different Definitions of Febrile Neutropenia

A variety of definitions of FN were used to construct the cohorts analyzed in the reviewed articles, differing in both fever temperature thresholds and ANC criteria for neutropenia. In [16], Klasterky et al. introduced the MASCC score with a definition of FN as ANC ≤ 500 and temperature ≥ 38 °C. In [17], Carmona-Bayonas et al. introduce the CISNE score with a definition of FN as ANC ≤ 500 (or ≤1000 with a predicted decrease to ≤500) and temperature ≥ 38 °C (Table 3).

The highest ANC for a patient to be neutropenic is presented in the paper by Gunderson et al. [38], where an ANC of ≤ 1500 cells/mm^3^ is used, and fever is defined as a temperature of 38.0 °C for one hour, or a single measurement of 38.3 °C.

It is seen that the only [28,34,37,38,40] which are studies that use the expected increase in temperature are the papers that define fever as 38.3 °C, whereas all other papers define it as 38.0 °C without the need for an increase in temperature.

### 3.3. Augmentation of Scores

In [42], Virizuela et al. discussed that MASCC fails to accurately predict patients with low risk of complications. To combat this five of the studies propose alterations to the MASCC score [29,31,39,40,41]. The authors of [29] analyzed the predictive performance of PCT levels. If a patient had a PCT level of ≥0.25 ng/mL they would be considered high risk. The authors concluded that PCT is a valuable adjunct for FN risk stratification and may enhance the predictive performance of the MASCC score. This is further analyzed in [31], where PCT was added as a measure to the low-risk group as stratified by the MASCC score. If a patient in the low-risk group has a PCT level < 0.5 ng/mL, the group was further categorized into high- and low-risk for stratification of either septic shock or bacteremia. This study has the limitation of being non-validated and is a single-center study; however, it shows that including PCT as an adjunctive biomarker along with MASCC could improve the stratification into low- and high-risk groups [31]. The same analysis was also conducted in a more recent paper [41], where the authors show that using PCT for predicting mortality and bacteremia in patients had a larger AUC than using the MASCC. Furthermore, in [41], a cutoff for PCT of 1.42 ng/mL was added to the low-risk FN patients, given from the MASCC score. From this, it was shown that with a PCT level ≥ 1.42 ng/mL had a mortality rate of 48%, highlighting the importance of adding PCT as a risk stratification metric.

Compared to [31], the authors in [41] conducted two separate analyzes for bacteremia and mortality. Both [31,41] show the advantages of adding a PCT-based stratification to low-risk patients, as a method to further classify low- and high-risk patients. An increase in predictive values by adding PCT to MASCC was also found in [40]. Furthermore, the authors also added lipopolysaccharide (LBP) as a predictive marker based on its similar diagnostic accuracy to PCT. Both features proved to improve accuracy when risk stratifying for serious complications. In [39], an improvement to the MASCC was also proposed, where the patient’s reported outcome was included. This adds features like feeling, function, and perception of health-related quality of life. This study showed suboptimal results, which were concluded by the authors to be caused by the administration of the PRO questionnaire among other factors.

### 3.4. Different Definition of Complications

In [16], along with the introduction of the MASCC score, a list of “poor outcomes” that define high-risk patients was also introduced, and this can also be found in Supplementary Material File S1. Most of the studies included in this review have either adapted this list or revised it. In [32,34], this list is used unchanged to define a serious complication, while [40] adapted the list to also include the presence of bacteremia. And with a more significant change to the list, [39] also added, death before fever resolution, hospital readmission, sepsis, septic shock, and a positive blood culture test.

In the studies focusing on gynecological patients, the list from [16] was applied with additional criteria [28,37,38]. In [37] in-hospital death was added, whereas in [28,38] the list was expanded with the inclusion of death within 14 days of ICU discharge, fungal infections, and allergic reactions.

Despite the application of the MASCC score in [30,33,35,36], the list of complications introduced with [16] is not applied. Instead, ref. [36] uses 30-day mortality to define serious complications, and [35] expands this by also including positive blood culture and sepsis-induced hypotension. In [30], the primary outcome is defined as sepsis, with a secondary outcome of ICU admission and 28-day mortality. In [33] a more non-quantitative approach was taken, based on patient function and degree of discomfort.

In [29,31,40,41] the performance of PCT as a predictive marker for risk stratification of FN was analyzed. Although these papers also introduced varying definitions of serious complications, none utilized the list introduced in [16]. In [29], the definition of serious complication was split into two outcomes: the primary outcome being in-hospital mortality, and the secondary outcome being admission to the ICU. In [31] the primary outcome was defined as septic shock or bacteremia. Finally, in [41], a non-quantitative definition, identical to the one used in [33] is employed.

### 3.5. Retrospective Studies

To assess and obtain outcomes based on the MASCC and CISNE, it is necessary to regularly record the scores or, as demonstrated in all the papers reviewed, except for [35,37], to incorporate them as part of a prospective study. This is caused by parts of the scores being subjective for the specific doctor handling the patient. This is noted in [41], where the PCT performance in risk-stratifying FN is analyzed. The MASCC score was determined retrospectively in [34,37] by a study of patient journals. A medical student examined case charts in [31], and the MASCC score was computed from this information. The inclusion of the subjective components of the MASCC, namely the burden of disease and the presence of dehydration requiring IV fluids was flagged as being “problematic”. Therefore, a set of regulations was established to delineate these elements, contingent upon the doctor’s inclusion of them in the journal. Both CISNE and MASCC were retrospectively calculated in [37]. Here it is stated that all components of the CISNE and MASCC scores were calculated; however, the details on how the scores were calculated were not presented.

## 4. Discussion

With the introduction of risk stratification scores, medical personnel have been provided with tools to rapidly classify patients with FN into risk categories that can assist with early discharge or treatment intensity. The present scoping review demonstrates that even though MASCC and CISNE scores dominate the field, their application across studies is characterized by substantial methodological heterogeneity. This heterogeneity is not to be interpreted as a lack of understanding or agreements within the field, but rather a reflection of the field and the ongoing efforts to improve predictive accuracy, importantly in regard to increasing the performance on low-risk classified patients.

One of the key findings of this review is that a common variation across the studies is the definition of FN. Although some studies adhere to the established definitions associated with MASCC and CISNE, variations exist in temperature thresholds for fever, ANC thresholds for neutropenia and requirements for sustained versus single measurements. There is nothing indicating a context specific adaption, but they could indicate adaptions to increase performance and adaptability instead of a general conflict of definition. Nevertheless, this variability has implications for general cohort homogeneity and consequently for the interpretation and comparability of performance.

Differences in FN definitions are also likely to influence the incidence of complications across the studies. Broader definitions, such as a higher ANC threshold or lower temperature threshold for fever, may include patients with less profound immunosuppression, thereby diluting complication rates in the low-risk groups. Conversely, stricter definitions might falsely enrich the cohorts for high-risk patients. These variations complicate the direct comparison of complication rates and predictive performance across studies using the same predictive tools. And this might also partially explain why a large proportion of low-risk patients as classified by the MASCC score still experience serious outcomes.

Several studies attempted to augment the MASCC score by including different features, e.g., PCT levels for the patient in order to improve performance on low-risk patients. The papers found enough of an improvement to suggest that further research is required and beneficial in this field and could return promising results. Furthermore, this indicates that there exist improvements, especially to the MASCC score, and potentially also the CISNE score. However, with classical methods like these, the variables used for the prediction are limited to the clinicopathologic features made available at the time of stratification. Another limitation of these scores is the inclusion of subjective features which can be beneficial due to the inclusion of expert opinions from medical personnel into the score; however, it can also introduce human errors, which scores like these should combat. Furthermore, it makes it extremely difficult to retrospectively validate the scores if the subjective features have not been noted as part of the hospital routine. If this is not the case, the scores need to be calculated and evaluated retrospectively, which can be prone to errors. Machine learning methodologies have been introduced in this field, albeit in other settings. These have been shown to achieve great performance in predicting the risk of patients by including a vast number of features [43].

A limitation mentioned in many of the papers is the modest size of the cohort. A consequence of this is a shift in the use of the scores presented, as they will be applied on varying cohorts, and therefore also with a limited resolution of the high- and low-risk patients, as well as the complications that are defined in the papers.

A limitation of the present work is related to the search strategy used in the review. The literature search was restricted to PubMed and Scopus databases, utilizing “risk stratification” as the primary search term. While this approach ensured that the retrieved literature explicitly addressed the use of risk stratification in febrile neutropenia, it may have limited the retrieval and excluded relevant studies. Further work could explore a broader search strategy. Furthermore, although the work focused on the use of risk stratification methods in adults, a few included studies utilized patients younger than 18 years of age. These studies were included when the patients were treated with adult oncology treatments, thereby reflecting the utilization of the methods in a general oncological setting rather than patient age. This method was used as some studies utilized mixed cohorts in terms of age, but applied adult treatment options, which was relevant for the objective of this review.

There exist papers that cover the performance of risk stratification scores for FN [44], but to the best of the authors’ knowledge, this is the first paper that investigates the inconsistencies in usage and how it affects the research. Collectively, the reviewed literature suggests that the primary challenge in FN risk stratification is not the absence of validated tools, but rather a need for layered approaches that can help improve performance in low-risk patients. Emerging data-driven methods including machine learning based models may offer future opportunities by incorporating a broader range of variables and reduce the reliance on subjective assessments. While such approaches are not yet readily available, they represent a logical extension of the current efforts to improve predictive performance.

## 5. Conclusions

This scoping review identified substantial methodological heterogeneity in the application of risk stratification scores for FN, with the definitions of FN and serious outcomes. Rather than implicating a lack of general consensus and understanding, this variability would appear to be a deliberate attempt to improve usability and performance of the existing scores. While such heterogeneity limits the direct comparability of the performance of models in the reviewed works, it indicates the fields progression towards more refined risk stratification tools.

Therefore, future research would greatly benefit from standardized reporting of FN and outcome definitions to facilitate benchmarking and external validation. Continued methodological innovation, including the implementation of data-driven models, may further enhance the safety and clinical usability of FN risk stratification.

## Figures and Tables

**Table 1:** List of the population, intervention, comparison and study (PICOS) inclusion criteria.

PICOS Components and Other Inclusion Criteria	Inclusion Criteria
P (population)	Adult participants diagnosed with FN.
I (intervention)	Scoring methods for FN risk stratification.
C (comparison)	Hybrid and other classical scoring methods for FN risk stratification.
O (outcomes)	Applying classical or hybrid risk stratification scores to predict low-risk or high-risk FN patients.
S (study design)	Clinical trials, quasi-experimental trial, randomized clinical trial (RCT), non-RCT, observational studies, retrospective cohorts, real world data and epidemiological studies.
Language	Publications in English.
Time	Publications between 2000 and 2024.
Document type	Articles.

**Table 2:** Results of included studies.

Reference	Cohort Size	Age and Gender	Methods	Type of Cancer(s)	Definition of Fever	Definition of Neutropenic	Definition of Primary Outcome	Setting
Gunderson et al. [28]	83 patients	Age: Mean 62 years (range 25–85) Male: 0% Female: 100%	MASCC	Gynecologic (100%)	One time reading: 38.3 °C Measurement with expected increase: 38.0 °C	ANC at admission: 1500 ANC with expected decrease: Not utilized	Primary outcome: As defined by [16] including death within 14 days of hospital discharge, fungal infection, allergic reaction.	Single center
Coyne et al. [29]	4434 patients	Age: Mean 57.38 years Median 59 years Male: 59.3% Female: 40.7%	Procalcitonin (PCT) Level	Hematologic and solid tumors (distribution not presented)	One time reading: 38.0 °C Measurement with expected increase: Not utilized	ANC at admission: 1000 ANC with expected decrease: Not utilized	Primary outcome: in-hospital mortality Secondary outcome: admission to ICU.	Multicenter
Kim et al. [30]	615 patients	Age: Mean ± SD 54.3 ± 13.8 years Male: 33.2% Female: 66.8%	MASCC, qSOFA and SIRS	Hematologic (20.8%) and solid tumors (79.2%)	One time reading: 38.0 °C Measurement with expected increase: Not utilized	ANC at admission: 500 ANC with expected decrease: 1000	Primary outcome: sepsis (presence of infection, together with its systemic manifestations) Secondary outcome: ICU admission and 28-day mortality	Single center
Ahn et al. [31]	355 patients	Age: Mean ± SD 54.0 ±14.2 years (range 17–86) Male: 36.9% Female: 63.1%	PCT level, as well as MASCC with PCT	Hematologic (19.2%) and solid tumors (80.8%)	One time reading: 38.0 °C Measurement with expected increase: Not utilized	ANC at admission: 500 ANC with expected decrease: 1000	Septic shock and bacteremia are the primary outcomes.	Single center
Taj et al. [32]	226 patients	Age: Mean 20 years (range 3–81) Male: 76.4% Female: 23.4	MASCC	Hematologic (100%)	One time reading: 38.0 °C Measurement with expected increase: Not utilized	ANC at admission: 500 ANC with expected decrease: 1000	Primary outcome: As defined by [16].	Not mentioned
Coyne et al. [33]	230 Patients	Age: Mean 55 years (range 21–86) Male: 49.1% Female: 50.9%	MASCC and CISNE	Hematologic (65.7%) and solid tumors (34.3%)	One time reading: 38.0 °C Measurement with expected increase: Not utilized	ANC at admission: 1000 ANC with expected decrease: Not utilized	Primary outcome: “those that caused severe discomfort or severely limited functioning and the performance of daily activities” Secondary outcome: “those that made the patients uncomfortable and had a negative influence on the performance of daily activities”	Not mentioned
Kumar et al. [34]	159 patients	Age: Median 30 years (range 16–65) Male: 63.5% Female: 36.5%	MASCC at cutoff ≥21 and ≥18	Hematologic (87.7%) and solid tumors (12.3%)	One time reading: 38.3 °C Measurement with expected increase: 38.0 °C	ANC at admission: 500 ANC with expected decrease: 1000	Primary outcome: As defined by [16].	Single center (or not mentioned)
Baugh et al. [35]	173 patients	Low risk: Age: Median 58 years (IQR 47–66) High risk: Age: Median 61 years (IQR 49–67) Male: 57.2% Female: 42.8%	MASCC	Hematologic (46.9%) and solid tumors (53.1%)	One time reading: 38.0 °C Measurement with expected increase: Not utilized	ANC at admission: 1000 ANC with expected decrease: Not utilized	Primary outcome: Identification of bacteremia or fungemia (positive blood culture), sepsis-induced hypotension or death within 30 days of emergency department (ED) visit.	Single center
Mohindra et al. [36]	129 patients	Age: Median 28 years (IQR 18–45) Male: 66.67% Female: 33.33%	CISNE and MASCC	Hematologic (90.6%) and solid tumors (9.4%)	One time reading: 38.0 °C Measurement with expected increase: Not utilized	ANC at admission: 1000 ANC with expected decrease: Not utilized	Primary outcome: 30-day mortality.	Single center
Monuszko et al. [37]	50 patients	Age: Mean ± SD 60 ± 15 years Male: 0% Female: 100%	MASCC and CISNE retrospectively	Gynecologic (100%)	One time reading: 38.3 °C Measurement with expected increase: 38.0 °C	ANC at admission: 1000 ANC with expected decrease: Not utilized	Primary outcome: As defined by [16] including in-hospital death.	Single center
Gunderson et al. [38]	31 Patients	Age: Median 63 years (range 47–77) Male: 0% Female: 100%	MASCC	Gynecologic (100%)	One time reading: 38.3 °C Measurement with expected increase: 38.0 °C	ANC at admission: 1500 ANC with expected decrease: Not utilized	Primary outcome: As defined by [16] including death within 14 days of hospital discharge, fungal infection, allergic reaction.	Multi center
Wang et al. [39]	120 Patients	Age: Mean ± SD 54.8 ± 12.6 years Male: 31.7% Female: 68.3%	MASCC and PROMASCC	Hematologic (26.7%) and solid tumors (73.3%)	One time reading: 38.0 °C Measurement with expected increase: Not utilized	ANC at admission: 500 ANC with expected decrease: Not Utilized	Primary outcome: As defined by [16] in addition to death before fever resolution, the persistence of positive blood cultures or breakthrough bacteremia, proven invasive or superficial fungal infection, allergic reaction, hospital readmission before complete fever resolution, sepsis, or septic shock.	Single center
De Guadiana-Romualdo et al. [40]	102 patients	Age: Median 63 years (range 21–85) Male: 39% Female: 61%	MASCC, PCT and LBP	Hematologic (77%) and solid tumors (23%)	One time reading: 38.0 °C Measurement with expected increase: Not utilized	ANC at admission: 500 ANC with expected decrease: Not Utilized	Primary outcome: As defined by [16] Secondary outcome: Bacteremia	Single center
Yadav et al. [41]	100 patients	Age: Mean ± SD 30.22 ± 14.72 years (range 13–61) Male: 70% Female: 30%	MASCC and PCT	Hematologic (8%) and solid tumors (92%)	One time reading: 38.0 °C Measurement with expected increase: Not utilized	ANC at admission: 1000 ANC with expected decrease: Not Utilized	Primary outcome: “Those that caused severe discomfort or severely limited functioning and performance of daily activities” Secondary outcome: “Those that made the patient uncomfortable and negatively affected their daily activities”	Single center

**Table 3:** Definitions used for fever and neutropenia.

Reference	Single ≥ 38.3 °C	Single ≥ 38.0 °C	Sustained ≥ 38.0 °C	ANC ≤ 500	ANC ≤ 1000	ANC ≤ 1500	Expected ANC Decline
Gunderson et al. [28]	✓	-	✓	-	-	✓	-
Coyne et al. [29]	-	✓	-	-	✓	-	-
Kim et al. [30]	-	✓	-	✓	-	-	✓
Ahn et al. [31]	-	✓	-	✓	-	-	✓
Taj et al. [32]	-	✓	-	✓	-	-	✓
Coyne et al. [33]	-	✓	-	-	✓	-	-
Kumar et al. [34]	✓	-	✓	✓	-	-	✓
Baugh et al. [35]	-	✓	-	-	✓	-	-
Mohindra et al. [36]	-	✓	-	-	✓	-	-
Monuszko et al. [37]	✓	-	✓	-	✓	-	-
Gunderson et al. [38]	✓	-	✓	-	-	✓	-
Wang et al. [39]	-	✓	-	✓	-	-	-
De Guadiana-Romualdo et al. [40]	-	✓	-	✓	-	-	-
Yadav et al. [41]	-	✓	-	-	✓	-	-

## Data Availability

Data sharing is not applicable to this article as no new data were created or analyzed in this study.

## Supplementary Materials

The following supporting information can be downloaded at: https://www.mdpi.com/article/10.3390/cancers18060987/s1, Supplementary Material File S1: Description of “poor outcomes” as defined in The Multinational Association for Supportive Care in Cancer risk index.
