# Supplementary material for: Use of Scores in Risk Stratification of Febrile Neutropenia—A Scoping Review
Source: Cancers (Basel). 2026 Mar 18;18(6):987. doi: 10.3390/cancers18060987 (PMC13024589; doi:10.3390/cancers18060987)
Supplement: Supplementary file 1 [file cancers-18-00987-s001.zip › cancers-4160386-supplementary.pdf]

Supplementary Material File S1

Poor outcomes as defined by The Multinational Association for Supportive Care in Cancer risk index:

- Hypotension: systolic blood pressure <90 mmHg or need for pressor support to maintain BP.
- Respiratory failure: PaO_2 <60 mmHg while breathing room air, or need for mechanical ventilation.
- ICU admission.
- Disseminated intravascular coagulation.
- Confusion or altered mental state.
- CHF seen on chest x-ray and requiring treatment.
- Bleeding severe enough to require transfusion.
- Arrhythmia or EKG changes requiring treatment.
- Renal failure requiring investigation and/or treatment with IV fluids, dialysis, or any other intervention.
- Other complications judged serious and clinically significant by the investigator.
